# Effects of manual lymphatic drainage on breast cancer-related lymphedema: a systematic review and meta-analysis of randomized controlled trials

**DOI:** 10.1186/1477-7819-11-15

**Published:** 2013-01-24

**Authors:** Tsai-Wei Huang, Sung-Hui Tseng, Chia-Chin Lin, Chyi-Huey Bai, Ching-Shyang Chen, Chin-Sheng Hung, Chih-Hsiung Wu, Ka-Wai Tam

**Affiliations:** 1 Department of Nursing, College of Medicine and Nursing, HungKuang University, Taichung City, Taiwan; 2 Department of Physical Medicine and Rehabilitation, Taipei Medical University Hospital, Taipei, Taiwan; 3 School of Nursing, Taipei Medical University, Taipei, Taiwan; 4 School of Public Health, Taipei Medical University, Taipei, Taiwan; 5 Division of General Surgery, Department of Surgery, Taipei Medical University Hospital, Taipei, Taiwan; 6 Division of General Surgery, Department of Surgery, Taipei Medical University - Shuang Ho Hospital, Taipei, Taiwan; 7 Department of Surgery, School of Medicine, College of Medicine, Taipei Medical University, Taipei, Taiwan; 8 Graduate Institute of Clinical Medicine, College of Medicine, Taipei Medical University, Taipei, Taiwan; 9 Center for Evidence-Based Medicine, Taipei Medical University, Taipei, Taiwan

**Keywords:** Manual lymph drainage, Lymphedema, Breast cancer, Meta-analysis

## Abstract

**Background:**

Lymphedema is a common complication of axillary dissection for breast cancer. We investigated whether manual lymphatic drainage (MLD) could prevent or manage limb edema in women after breast-cancer surgery.

**Methods:**

We performed a systematic review and meta-analysis of published randomized controlled trials (RCTs) to evaluate the effectiveness of MLD in the prevention and treatment of breast-cancer-related lymphedema. The PubMed, EMBASE, CINAHL, Physiotherapy Evidence Database (PEDro), SCOPUS, and Cochrane Central Register of Controlled Trials electronic databases were searched for articles on MLD published before December 2012, with no language restrictions. The primary outcome for prevention was the incidence of postoperative lymphedema. The outcome for management of lymphedema was a reduction in edema volume.

**Results:**

In total, 10 RCTs with 566 patients were identified. Two studies evaluating the preventive outcome of MLD found no significant difference in the incidence of lymphedema between the MLD and standard treatment groups, with a risk ratio of 0.63 and a 95% confidence interval (CI) of 0.14 to 2.82. Seven studies assessed the reduction in arm volume, and found no significant difference between the MLD and standard treatment groups, with a weighted mean difference of 75.12 (95% CI, −9.34 to 159.58).

**Conclusions:**

The current evidence from RCTs does not support the use of MLD in preventing or treating lymphedema. However, clinical and statistical inconsistencies between the various studies confounded our evaluation of the effect of MLD on breast-cancer-related lymphedema.

## Background

Lymphedema is defined as persistent tissue swelling caused by the blockage or absence of lymph drainage [1]. Lymphedema is a major concern for patients undergoing axillary lymph-node dissection for the treatment of breast cancer. The incidence of lymphedema at 12 months after breast surgery ranges from 12% to 26% [2,3]. Lymphedema may result in cosmetic deformity, loss of function, physical discomfort, recurrent episodes of erysipelas ,and psychological distress [4,5]. Thus, an effective treatment for lymphedema is necessary.

Previous surgical techniques for the treatment of lymphedema aimed to reduce limb volume using a debulking resection approach. With the advent of microsurgery, use of multiple lymphatic-venous anastomoses has become the most common surgical treatment [6]. However, convincing evidence of the success of lymphatic-venous anastomoses has not been demonstrated. Thus, most patients with lymphedema choose non-surgical treatments, such as the use of elastic stockings, especially in early stages of lymphedema [7].

Complex decongestive physiotherapy (CDP) is likely to reduce upper limb lymphedema in patients with breast cancer. Evidence of the efficacy of other physiotherapy methods is limited [8-10]. Compression bandaging, manual lymphatic drainage (MLD), physical exercise to maintain lymphatic flow, and skin care are combined in CDP [11,12]. In MLD, specialized rhythmic pumping techniques are used to massage the affected area and enhance the lymph flow. Gentle skin massage is thought to cause superficial lymphatic contraction, thereby increasing lymph drainage [13].Vodder originally suggested the use of range-of-motion exercises to relieve various types of chronic edema, such as sinus congestion and catarrh [14], and the use of MLD has become a common treatment for lymphedema worldwide, especially in European hospitals and clinics.

To date, several studies have been published investigating the effects of MLD in preventing and treating lymphedema after breast-cancer surgery [15-18]. However, these studies have been inconclusive, probably because of small sample sizes. Therefore, we conducted a systematic literature review and meta-analysis of randomized controlled trials (RCTs) to evaluate the effectiveness of MLD in the prevention and treatment of breast-cancer-related lymphedema.

## Methods

### Selection criteria

We reviewed RCTs or quasi-RCTs from the literature that evaluated the outcome of MLD in preventing and treating breast-cancer-related lymphedema. For inclusion in our study, the trials were required to describe: 1) the inclusion and exclusion criteria used for patient selection, 2) the MLD technique used, 3) the compression strategy used, 4) the definition of lymphedema, and 5) the evaluation of lymphedema severity. We excluded trials that met as least one of the following criteria: 1) patients had not received axillary lymph-node dissection (such as in studies in which only sentinel node sampling was used), 2) the clinical outcomes had not been clearly stated, or 3) duplicate reporting of patient cohorts had occurred.

### Search strategy and study selection

Studies were identified by keyword searches of the following electronic databases: PubMed, EMBASE, CINAHL, PEDro (Physiotherapy Evidence Database), SCOPUS, Cochrane Central Register of Controlled Trials, and the ClinicalTrials.gov registry (http://clinicaltrials.gov/). The following terms and Boolean operator were used in MeSH and free-text searches: ‘manual lymph drainage’, ‘breast cancer OR neoplasm’, ‘lymphoedema OR lymphedema’. The ‘related articles’ facility in PubMed was used to broaden the search. No language restrictions were applied. The final search was performed in December 2012. We attempted to identify additional studies by searching the reference sections of any relevant papers and contacting known experts in the field.

### Data extraction and methodological quality appraisal

Two authors (K-WT and T-WH) independently extracted details of the RCTs pertaining to the participants, inclusion and exclusion criteria, manual lymph-drainage techniques used, arm lymphedema parameters, and complications. The individually recorded decisions of the two reviewers were compared, and any disagreements were resolved based on the evaluation of a third reviewer (S-HT).

The two authors independently appraised the methodological quality of each study based on: 1) adequacy of the randomization, 2) allocation concealment, 3) blinding, 4) duration of follow-up, 5) number of drop-outs, and 6) performance of an intention-to-treat (ITT) analysis.

### Outcomes assessments

The efficacy of MLD was evaluated by the incidence of lymphedema and the reduction in the volume of the patient’s arm at 1, 3, 6, 9 and 12 months after MLD treatment. The arm volume was assessed by submerging each arm in a container filled with water, and measuring the volume (ml) displaced [19]. The absolute edema volume was defined as the difference in volume between the arm with lymphedema and the contralateral arm [18]. The following various definitions for lymphedema were used in the studies analyzed: a difference in volume of greater than 10% between the affected arm and contralateral arm [17,18,20]; an increase of 200 ml or more in the volume of the affected arm compared with the pre-surgery volume of the same arm [16]; and an increase of 20 mm or more in the circumference of the affected arm compared with the pre-surgery circumference of the same arm [16,21].

### Statistical analysis

Statistical analysis was conducted using Review Manager software (version 5.1;Cochrane Collaboration, Oxford, UK). The meta-analysis was performed in accordance with the Preferred Reporting Items for Systematic Reviews and Meta-analysis (PRISMA) guidelines [22]. When necessary, standard deviations (SDs) were estimated based on the reported confidence interval (CI) limits, standard error, or range values [23]. The effect sizes of dichotomous outcomes were calculated as risk ratios (RR), and the mean difference was calculated for continuous outcomes. The precision of an effect size was calculated as the 95% CI. A pooled estimate of the RR was calculated using the DerSimonian and Laird random-effects model [24]. This provided relatively wide CIs and an appropriate estimate of the average treatment effect for trials that were statistically heterogeneous, resulting in a conservative statistical claim. The data were pooled only for studies that exhibited adequate clinical and methodological similarity. Statistical heterogeneity was assessed using the *I*^2^ test, with *I*^2^ quantifying the proportion of the total outcome variability that was attributable to variability among the studies.

## Results

### Characteristics of the trials

**Figure 1:**
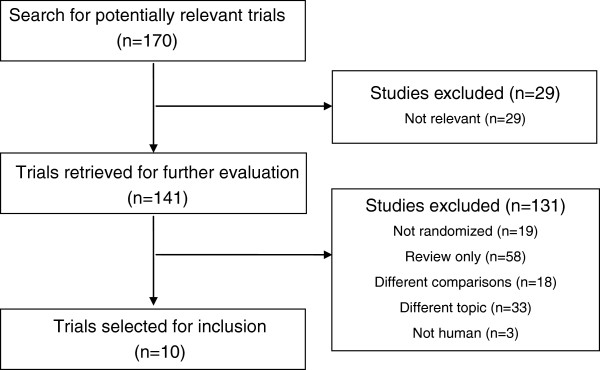
Flowchart of the selection of the clinical trials.

The process by which we screened and selected the trials is shown in a flow chart (Figure 1). Our initial search yielded 170 studies, of which 29 were deemed ineligible after screening of titles and abstracts. Another 141 reports were excluded from our final analysis for the following reasons: 58 were review articles, 3 were animal studies, 18 had used different comparisons, 33 discussed different topics, and 19 were not randomized trials. The remaining 10 eligible RCTs [15-18,20,21,25-28] were included in our analysis.

The 10 trials were published between 1998 and 2011, and had sample sizes ranging from 24 to 158 patients (Table 1). All patients had undergone mastectomy with axillary lymph-node dissection, and patient age ranged from 25 to 77 years.

**Table 1:** Characteristics of studies that fulfilled the inclusion criteria for meta-analysis

**Reference**	**Inclusion criteria**	**No. of patients**	**Age, years, (mean ± SD)**	**Intervention**
**Treatment**				
Andersen, 2000	Symptoms of lymphedema; 20 mm circumference or 200 ml volume difference between arms	C: 22	C: 56 (29 to 77)^a^	C: Sleeve and glove compression 32 to 40 mmHg + exercises + skin care + safety precautions
		I: 20	I: 53 (25 to 73)	I: C + MLD 8 times in 2 weeks
Didem, 2005	2-50 mm circumference difference between arms; lymphedema > 12 months after surgery	C: 26	C: 54.7 ± 12.1	C: Bandaging; elevation; head, neck and shoulder exercise, 3 days/week for 4 weeks
		I: 27	I: 53.1 ± 3.05	I: C + MLD
Johansson, 1998	>10% volume difference between arms	C: 12	C: 57.5 (47.5-69.5)^a^	C: Sleeve compression for 2 weeks + SPC 40 to 60 mmHg 2 hours/day for 2 weeks
		I: 12	I: 64 (52.5-69.5)	I: Sleeve compression 2 weeks + MLD for 5 days/weeks for 2 weeks
Johansson, 1999	>10% volume difference between arms	C: 18	C: 64 ± 12	C: Bandage compression for 3 weeks
		I: 20	I: 58 ± 12	I: C + MLD applied for 5 days during final weeks
McNeely, 2004	150 ml volume difference between arms	C: 24	C: 58 ± 13	C: Bandage compression for 4 weeks
		I: 21	I: 63 ± 13	I: C + MLD for 45 minutes/day 5 days/week for 4 weeks
Sitzia, 2002	Lymphedema of one arm secondary to treatment for breast cancer	C: 13	C: 75 ± 10.2	C: SLD 30 minutes/day, 5 days/weeks for 2 weeks + bandage + exercises
		I: 15	I: 68 ± 10.4	I: MLD 90 minutes/day, 5 days/week for 2 weeks + bandage + exercises
Williams, 2002	>10% volume difference between arms	G1: 15	A: 63 ± 13	G1: MLD for 3 weeks, then no treatment for 6 weeks, then SLD for 3 weeks
		G2: 16	B: 58 ± 13	G2: SLD for 3 weeks, then no treatment for 6 weeks, then MLD for 3 weeks
Szolnoky, 2009	Lymphedema >12 months after surgery	G1: 13	G1: 54.8	G1: MLD 60 minutes/day, 5 days/week for 2 weeks
		G2: 14	G2: 56.6	G2: MLD for 30 minutes/day then SPC 50 mmHg for 30 minutes/day, 5 days/week for 2 weeks
**Prevention**				
Devoogdt, 2011	Patients after breast-cancer surgery	C: 81	C: 54.5 ± 11.1	C: Exercise therapy 30 minutes/session
		I: 77	I: 55.8 ± 12.5	I: C + MLD 30 minutes/session for 40 sessions
Torres Lacomba, 2010	Patients after breast-cancer surgery	C: 60	C: 52.9 ± 12.5	C: Educational strategy
		I: 60	I: 52.9 ± 10.7	I: C + MLD + massage + exercise

Abbreviations: C, control; I, intervention; G, group; MLD, manual lymphatic drainage; SLD, simple lymph drainage; SPC, sequential pneumatic compression.

Values are mean ± standard deviation, except for ^a^mean (range).

Most of the trials had assessed MLD treatment using the Vodder method [14]. MLD was performed by specially trained physiotherapists, and was followed by skin care with moisturizers, multilayered short-stretch bandaging with appropriate padding, and exercise. The MLD extended to the neck, the anterior and posterior trunk, and the swollen arm. One study did not fully describe the MLD method that was used [15]. In addition to the use of sleeve or glove compression, standard therapies also included educational information and recommendations on lymphedema, instructions for physical exercises to enhance lymph flow, education in skin care, and safety precautions. Across all ten studies, the two treatment groups were comparable for patient age and the duration of MLD (Table 1). Most of the included trials had investigated whether the addition of MLD to the standard therapy after breast-cancer treatment improved clinical outcomes in women with lymphedema. Two trials investigated the preventive effect of MLD on the development of lymphedema in women after breast-cancer surgery [16,21]. Two trials measured the effects of simple lymphatic drainage (SLD) versus MLD on lymphedema of the arm [20,27]. One trial compared the outcomes of MLD with or without sequential pneumatic compression (SPC) [28].

We assessed the methodological quality of the included trials (Table 2). Five studies reported acceptable methods of randomization [16,21,25-27], four trials described the method of allocation concealment [16,25-27] three studies reported the blinding of the outcome assessors [16,21,25], and one trial reported the blinding of the patients [26]. Three studies used an ITT analysis [15,16,28]. The number of patients lost to follow-up was acceptable at less than 20% in all 10 studies.

**Table 2:** Assessment of methodological quality of included trials

**Study**	**Study design**	**Data analysis**	**Allocation generation**	**Allocation concealment**	**Blinding**	**Lost to follow-up**
Andersen, 2000	RCT	ITT	Unclear	Unclear	None reported	9.5% at 12 months
Devoogdt, 2011	RCT	ITT	Adequate	Adequate	Assessor blinded	4% at 12 months
Didem, 2005	RCT	PP	Sealed envelopes	Adequate	Patient blinded	5.4% at 1 months
Johansson, 1998	RCT	PP	Unclear	Unclear	None reported	None
Johansson, 1999	RCT	PP	Inadequate	Unclear	None reported	None
McNeely, 2004	RCT	PP	Computer-generated	Adequate	Assessor blinded	11.1% at 1 months
Williams, 2002	RCT crossover	PP	Unclear	Unclear	None reported	None
Sitzia, 2002	RCT	PP	Computer-generated	Adequate	None reported	3.6% at 2 weeks
Szolnoky, 2009	RCT	ITT	Unclear	Unclear	None reported	None
Torres Lacomba, 2010	RCT	PP	Computer-generated	Unclear	Assessor blinded	3.3% at 12 months

Abbreviations: PP, per-protocol; RCT, randomized controlled trial; ITT, intention-to-treat.

### Incidence of lymphedema

**Figure 2:**
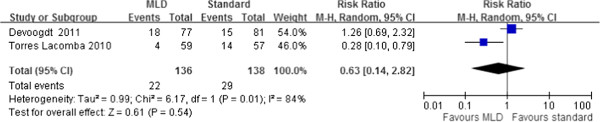
**Forest plot of the comparison of the effect of standard treatment with or without manual lymphatic drainage (MLD) on the incidence of post-mastectomy lymphedema from 2 clinical trials.** The first author names and the 95% confidence interval (CI) are included.

The incidence of lymphedema was determined in two trials that evaluated the preventive outcome of MLD in patients after breast-cancer surgery [16,21]. No significant differences were found between the MLD and standard treatment groups, with an RR of 0.63 (95% CI, 0.14 to 2.82) at 1 month [21] and 3 months [16,21] postoperatively (Figure 2). The value of *I*^2^ was 84%, indicating significant heterogeneity across the studies.

### Reduction in lymphedema volume

**Figure 3:**
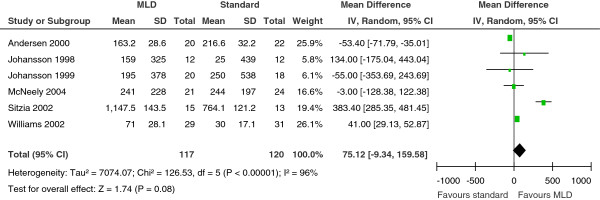
**Forest plot of comparison of the effect of compression therapy with or without manual lymphatic drainage (MLD) on the reduction in post-mastectomy lymphedema volume from 6 clinical trials.** The first author names, the standard deviations (SDs) of the mean, and the 95% confidence interval (CI) are included.

Seven studies provided data on the reduction in lymphedema volume [15,17,18,20,25,27,28] after MLD treatment. In each of these studies, the volume of the arm was measured at the beginning of treatment, and at 1, 3, and 12 months after treatment using water displacement volumetry. To facilitate our comparisons, we converted the percentage reductions in arm volume after MLD treatment to absolute volume (ml)reductions. Our analysis showed that there were no significant differences between the two treatment groups (weight mean difference 75.12; 95% CI −9.34 to 159.58), and that significant heterogeneity in the reductions in arm volume occurred between the trials (Figure 3).

The data reported by Didem *et al*. was not pooled because the method used to measure the change in lymphedema volume was not reported. However, that groups reported that lymphedema was more effectively reduced in the MLD treatment group than in the standard physiotherapy group (*P*<0.05) [26]. In addition, a study of the effects of MLD with or without SPC reported no significant difference in arm volume reduction between the treatment groups at 1 and 2 months after treatment [28].

## Discussion

A physical treatment program combining MLD, skin care, exercise, compression bandaging, and sleeve or stocking compression is recognized as providing optimal lymphedema management [29]. Three systematic reviews concluded that combined physical therapy provides effective treatment for lymphedema [30-32]. However, the effectiveness of the individual components of such programs has not been clearly established. The relatively high cost of MLD compared with compression bandaging warrants assessment of the efficacy of these individual components. The results of our systematic review and meta-analysis did not show a significant benefit for MLD in reducing lymphedema volume. Although individual studies reported advantages associated with MLD, methodological inconsistencies between the studies confounded our attempts to conduct an overall comparison of the effects of MLD across the studies.

The published reports of the effectiveness of MLD are conflicting. One prospective study of 682 individual cases in a single lymphology unit evaluated various treatments for lymphedema. The results indicated that the risk of failure for lymphedema therapy after intensive decongestive physiotherapy was primarily associated with younger age, higher weight, and higher body mass index. By contrast, elastic sleeve and multilayer bandaging treatments were associated with a reduced risk of treatment failure, whereas the use of MLD as an adjunct to those therapeutic components was not [33]. One retrospective study of 208 patients with lymphedema receiving palliative care showed clinical improvement in the intensity of pain and dyspnea in most patients after MLD treatment [34]. The advantage of the RCT design is that allocation bias is minimized, resulting in a balance between the known and unknown confounding variables in the assignment of treatments. Systematic review and meta-analysis of the clinical outcomes of therapy, as reported in the summaries of the RCT results to date, may help identify the effects that are common to these trials. Such research more clearly distinguishes the effects of MLD in preventing and managing lymphedema.

Our meta-analysis examined the results of six studies that assessed the effects of MLD in patients with post-mastectomy lymphedema, compared with compression therapy [15,17,18,20,25,27]. Compression bandaging has been shown to be effective in managing lymphedema. Badger *et al*. conducted an RCT to compare compression bandaging for 18 days followed by a compression garment (treatment group) versus the compression garment only (comparison group). These authors reported a significantly greater reduction in limb volume at 24 weeks in the treatment group compared with the comparison group [4]. The studies that we reviewed had investigated several types of compression therapy. McNeely *et al*. found that the figure-of-eight method was more effective in maintaining the correct bandage position, and was also more comfortable for the patient, compared with the spiral-bandaging method [25]. McNeely *et al*. replaced the bandages 5 times/week over the 4-week treatment period, whereas Johansson *et al*. replaced the compression bandage every 2 days over a 3-week period [18].

Sequential intermittent pneumatic compression is another nonsurgical treatment for lymphedema [35]. Szolnoky *et al*. investigated whether a combination of SPC treatments and MLD improved the outcome of CPD treatment for women with secondary lymphedema [28]. Thus, in the studies we investigated, there was a high level of heterogeneity regarding the variables measured to represent the reduction in lymphedema volume.

We included two studies in our analysis that compared MLD with SLD in the treatment of breast-cancer-related lymphedema [20,27]. Although MLD and SLD involve the same principles, SLD is a less complex technique that uses simplified hand movements in a set sequence. SLD can also be applied by the patient or a caregiver without requiring specialized training [27]. The results of both studies showed that MLD significantly reduced excess limb volume compared with SLD.

Of the ten RCT studies that we reviewed in our meta-analysis, only two investigated the effects of MLD for preventing lymphedema after breast-cancer surgery [16,21]. Devoogdt *et al*. evaluated the effect of MLD used in combination with exercise therapy and instructional guidelines for lymphedema prevention in 160 patients with breast cancer and unilateral axillary lymph-node dissection, who were stratified by body mass index and axillary irradiation [16]. Patients received exercise therapy plus MLD or exercise therapy only for 6 months; the results showed no significant difference in the prevention of lymphedema between the two groups [16]. By contrast, Torres Lacomba *et al*. used MLD, scar-tissue massage, and progressive active and action-assisted shoulder exercises postoperatively in patients who had undergone breast-cancer surgery, whereas their control group received only instructional guidelines for lymphedema prevention Torres Lacomba *et al*. found a significant difference in secondary lymphedema between the groups at 1 year post-surgery [21]. However, the individual contribution of MLD to the prevention of secondary lymphedema was unclear.

Variability in clinical factors and non-uniform reporting of clinical parameters contributed to the heterogeneity between the studies that we reviewed. First, the technique, duration, and frequency of MLD differed across the studies, and one study did not report the technical details of their MLD method [15]. Second, the experience of the physiotherapist and the characteristics of the individual patient can affect clinical outcomes. For example, patients in the study by Sitzia *et al*. were older than those in the other trials that we reviewed [27]. Third, the compression and exercise strategies also differed greatly between the studies that we reviewed (Table 1); for example, the control group in the study by Torres Lacomba *et al*. received only educational instructions [21]. Fourth, the methods used for evaluating the reduction in arm volume were also different between the studies, rendering our assessment vulnerable to measurement bias.

The strengths of our review include our comprehensive search for relevant studies, the systematic and explicit application of eligibility criteria, the careful consideration of study quality, and our rigorous analytical approach. However, our review was limited by the methodological quality of the original studies (Table 2). First, several trials were small, and one study recruited only 12 patients in each treatment group [17], diminishing the statistical power of their analysis. Second, only half of the studies included in our analysis reported adequate randomization in the study-group allocation [16,21,25-27]. Third, in seven studies, the assessment staff were not blinded to the outcomes [15,17,18,20,26-28]. Furthermore, most of the investigators analyzed their data according to the per-protocol principle, which may have biased their evaluations of the effect of MLD.

An ongoing study of 58 patients with post-mastectomy lymphedema is evaluating the effectiveness of MLD as an adjunct to standard treatment for reducing the volume of the affected arm and the consequent effects on patient quality of life and physical limitations [ClinicalTrials.gov identifier NCT01152099] [36]. We await the results to determine whether this will provide more evidence for clinical practice.

## Conclusion

In conclusion, our meta-analysis indicated that the addition of MLD to compression and exercise therapy for the treatment of lymphedema after axillary lymph-node dissection for breast cancer is unlikely to produce a significant reduction in the volume of the affected arm. We found no significant difference in the incidence of lymphedema in patients treated with or without MLD. Overall, the methodological quality of the studies that we reviewed was poor. Based on the results of our meta-analysis, we cannot recommend the addition of MLD to compression therapy for patients with breast-cancer-related lymphedema.

## Competing interests

The authors have no conflicts of interest or financial ties to disclose.

## Authors’ contributions

K-WT and T-WH devised the study. K-WT, T-WH, and S-HT extracted the data. K-WT, T-WH, C-CL, and C-HB analyzed and interpreted the data. K-WT and T-WH wrote the first draft. All authors contributed to subsequent versions, and approved the final article. K-WT is the corresponding author.
